# The Effect of Virtual Reality on Motor Anticipation and Hand Function in Patients with Subacute Stroke: A Randomized Trial on Movement-Related Potential

**DOI:** 10.1155/2022/7399995

**Published:** 2022-01-24

**Authors:** Ling Chen, Yi Chen, Wen Bin Fu, Dong Feng Huang, Wai Leung Ambrose Lo

**Affiliations:** ^1^Department of Acupuncture and Moxibustion, The Second Clinical College of Guangzhou University of Chinese Medicine, Guangzhou, China; ^2^Guangzhou University of Chinese Medicine, Guangzhou, China; ^3^Department of Rehabilitation, The First Affiliated Hospital, Sun Yat-sen University, China; ^4^Guangdong Engineering and Technology Research Center for Rehabilitation Medicine and Translation, Sun Yat-sen University, Guangzhou 510080, China; ^5^Department of Rehabilitation Medicine, The Seventh Affiliated Hospital, Sun Yat-sen University, Shenzhen 518107, China

## Abstract

**Background:**

Impaired cognitive ability to anticipate the required control for an upcoming task in patients with stroke may affect rehabilitation outcome. The cortical excitability of task-related motor anticipation for upper limb movement induced by virtual reality (VR) training remains unclear.

**Aims:**

To investigate the effect of VR training on the cortical excitability of motor anticipation when executing upper limb movement in patients with subacute stroke.

**Methods:**

A total of thirty-six stroke survivors with upper limb hemiparesis resulting from the first occurrence of stroke within 1 to 3 months were recruited. Participants were randomly allocated to the VR intervention group or conventional therapy group. Event-related potentials (ERPs) and electromyography (EMG) were used to simultaneously record the cortical excitability and muscle activities during palmar grasp motion. Outcome measures of the contingent negative variation (CNV) latency and amplitude, EMG reaction time, Upper Limb Fugl-Meyer Assessment (UL-FMA), Action Research Arm Test (ARAT), and National Institutes of Health Stroke Scale (NIHSS) were recorded pre- and postintervention. The between-group difference was analysed by mixed model ANOVA.

**Results:**

The EMG onset time of the paretic hand in the VR group was earlier than that observed in the control group (*t* = 2.174, *p* = 0.039) postintervention. CNV latency reduction postintervention was larger in the VR group than in the control group (*t* = 2.411, *p* = 0.021) during paretic hand movement. The reduction in CNV amplitude in the VR group was larger in the VR group than in the control group (*p* < 0.001 for all electrodes except for C3) when executing paretic hand movement. ARAT and UL-FMA scores were significantly higher in the VR group than in the control group (*p* = 0.019 and *p* = 0.037, respectively) postintervention. No significant difference in the reduction in NIHSS was found between the VR and control groups (*p* = 0.072).

**Conclusions:**

VR intervention is superior to conventional therapy to improve the cognitive neural process of motor anticipation and reduce the excessive compensatory activation of the contralesional hemisphere. The improvements observed in the cognitive neural process corroborated with the improvements in hand function.

## 1. Introduction

Stroke is one of the most severe issues encountered by the ageing population [1] as it is among the leading causes of long-term disability worldwide [2]. Published epidemiological study reported that approximately 60% of stroke survivors have motor dysfunction and 40% continue to have severe disability of the upper extremity [3]. Statistically, only 5% to 20% of stroke survivors recovered their upper limb function completely, 25% of them recover part of upper limb function, and 60% of them lost upper limb function completely [4]. Motor dysfunction of upper extremity contribute to reducing the ability to perform motor tasks such as reaching and grasping [5], which in turn affects the ability to perform activity of daily living such as eating, dressing, and washing [6, 7].

The clinical outcomes of upper extremity rehabilitation are influenced by cognitive function which directly affects the ability to acquire and execute functional skills [8, 9]. Motor anticipation is a key component of cognitive function which requires high level of the brain cognitive process [10]. It is one of the essential elements for successful motor execution since volitional movements are preceded by a period of planning, during which parameters are set for specific upcoming movement to achieve a specific goal [11]. Early literature on nonhuman primate reported increase in neural spike rate prior to movement initiation [12] which is considered a key signature of cognitive preparatory activity [11]. Study in human indicated that the brain activates 1.5 seconds before the execution of movements, while motor intention is perceived at 500 to 800 ms after the activation of the motor cortex [13]. On the other side, motor preparation requires the prediction of the physical properties of static objects and the dynamic loads generated by the objects in space [5, 14–16]. Studies in stroke survivors indicated that the ability to anticipate the required control to execute motor task is lacking [17] which contribute to motor intentionality and power accuracy out of control. Literature indicated that impaired anticipatory ability in stroke patients may present clinically as a marked increased grip force during object manipulation of the upper extremity [18–21].

The electrophysiological process that is associated with motor anticipation and whether changes in motor anticipation are associated with functional improvement are unclear in patients with stroke [22]. Existing studies indicated that a loss of frequency alpha band on the ipsilesional hemisphere was related to poor motor recovery outcome [23]. Improvement in coherence in the beta frequency band was reported to have linear relation with motor function improvement [24]. However, the frequency of alpha rhythm oscillations is associated with motor execution [25], and beta frequency is modulated during motor execution [26]. These electroencephalogram (EEG) components are suitable to assess neural activities during motor execution state but not the cognitive neural process of motor anticipation which takes place prior to the motor execution. Contingent negative variation (CNV) is a slow negative event-related potential (ERP) that can reflect the cognitive process of event-related motor preparation [27–29]. It is considered a reliable index for observing cortical excitability of motor anticipatory [22] and can be used to assess the cortical excitability during motor anticipation in stroke patients [30]. Study on source analysis indicated that CNV is generated from multiple sources, including cortical and subcortical generators of the anterior cingulate cortex, supplementary motor area, and primary motor area [27, 31, 32]. Stroke survivors with chronic hemiparesis demonstrated significantly enhanced latency and increased amplitude of late CNV at the midline during paretic and nonparetic hand preparation [27] which indicated greater anticipatory effort in response to task execution. This is given further support by a previous study which observed early CNV onset time with increased peak amplitudes of bilateral hemispheres in people with subacute stroke [31]. The study observed similar amount of computational demand from the contralesional and ipsilesional hemisphere during the motor planning phase, as reflected in the lack of significant difference in CNV latency and peak amplitude.

Virtual reality (VR) plays a prominent role in promoting functional recovery poststroke. It provides goal-orientated tasks and enriched motivational training to improve patients' ability to implement a planned motor task [32–34]. In addition, it has the potential to implement effective intervention at low cost [35]. A previously published meta-analysis identified 6 key principles of VR that are relevant to the neuroplasticity process. These principles include goal-oriented task, high number of repetition or training dosage, altering task difficulty, real-time feedback, increased users' motivation and engagement, and increased enjoyment of intensive task-relevant training [36]. Some of these principles are related to the motor planning process which may contribute to the improvement of upper limb motor function. The combination of exoskeletal support and VR was supposedly able to further promote the function recovery in patients with stroke [36]. A previous study utilised a combination of exoskeletal support and VR training to assess the impact on the motor planning process and gait function [37]. The study observed strong mu rhythm and event-related spectral perturbations post-VR intervention which were associated with clinical improvement in gait. This study offered some insight into the potential benefit of VR intervention on motor planning and subsequent functional recovery. To date, there is limited evidence on whether the cognitive neural process of motor planning may be improved by VR intervention and whether changes in the cognitive neural process may contribute to upper limb functional improvement. The aims of the present study were to investigate the impact of VR intervention on motor anticipation and upper limb function in people with subacute stroke. This study hypothesised that patients with stroke who underwent VR training had significant reduction in CNV latency and amplitude when executing palmar grasp movement with the paretic hand. Upper limb function would also improve significantly post-VR intervention. Improvements observed in the VR intervention group were significantly larger than the improvements observed in the conventional therapy group. A preprint of the article has previously been published [38].

## 2. Methods

### 2.1. Study Setting

This trial was a parallel randomized, single-blinded, controlled trial. Data collection took place in the Department of Rehabilitation, First Affiliated Hospital, Sun Yat-sen University.

### 2.2. Ethics

This study was approved by the Ethical Committee of the First Affiliated Hospital of Sun Yat-sen University (ethics approval number [2020]073). All participants were provided with a comprehensive explanation of the experimental procedure and a participant information sheet. Informed consent was obtained from all participants prior to study enrolment.

### 2.3. Recruitment and Sample Size

Recruitment took place between July 2016 and June 2018 in the inpatient ward of the Department of Rehabilitation Medicine, the First Affiliated Hospital of Sun Yat-sen University. The inclusion criteria were as follows: (1) first ever occurrence of stroke within 1 to 3 months, (2) stroke occurrence confirmed by magnetic resonance imaging (MRI) or computed tomography (CT), (3) age between 40 and 80 years, (4) having at least 20° of wrist flexion/extension and at least 10° of finger flexion and extension of the paretic limb, (5) able to sit for at least 30 minutes without assistance, and (6) no severe cognitive impairment (Mini‐Mental State Examination > 21) [39]. The exclusion criteria were as follows: (1) brainstem injury, (2) hand deformity, and (3) visual field deficits. Suitable participants were identified by the clinical team during the routine medical admission procedure. Patients were then approached by a member of the research team who was not involved in providing medical care to explain about the study. All approached patients were asked to contact a member of the research team to express their interest.

**Figure 1:**
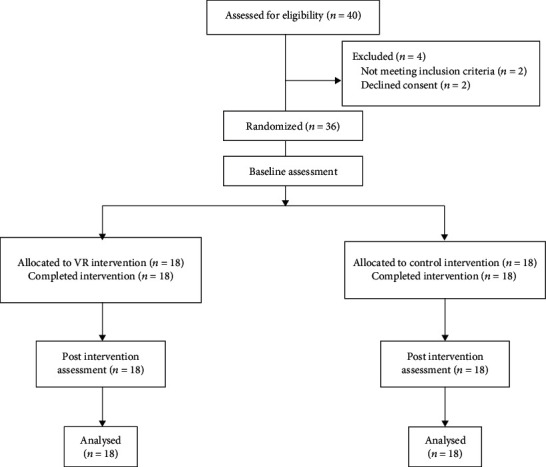
Flow diagram of the study.

Sample size was based on a pilot trial where 16 subacute stroke participants were allocated into VR and control groups. CNV latency during paretic hand movement postintervention was used as the primary outcome measure for sample size calculation. The calculation of sample size was conducted in the software GPower ver 3.1.2, using “A priori: Compute required sample size –given *α*, power, and effect size” as the type of power analysis. Preliminary results indicated a mean CNV latency of 1740.22 ms (SD 120.78) for the control group and 1614 ms (SD 122.61) for VR group postintervention, which gave an effect size of 0.50. With *α* error probability of 0.05 and a power of 0.95, a sample size of 36 was sufficient for the present study.  Figure 1 shows the number of participants at each stage of the study.

### 2.4. Randomization, Concealment, and Blinding

Participants were randomly allocated to either VR group or control group in a 1 : 1 ratio by simple randomization. The randomization schedule was calculated in the statistical software SPSS (IBM SPSS Statistics version 20, USA) by a statistical expert from the Faculty of Medical Statistics and Epidemiology, Sun Yat-sen University. The sequence of allocation was kept in sealed envelopes and revealed by a member of the research team after the participant was enrolled. Participants were blinded from their group allocation but were informed that they had an equal chance of allocation to the VR or control group before study participation. The outcome assessor was blinded to group allocation, but the treating therapists were not blinded.

### 2.5. Procedure

Interventions were delivered for 2 weeks with 5 training sessions per week. Each session lasted for 60 minutes. Designated therapists provided either VR training or occupational therapy for upper limb function in addition to routine medical care and other rehabilitation deemed necessary by treating physicians.

### 2.6. Outcome Measures

Outcome measures of behavioural data included EMG onset time and CNV latency. CNV peak amplitude was adopted to assess the cortical excitability during the motor planning phase. Clinical assessment scales of the Action Research Arm Test (ARAT) [40], Upper Limb Fugl-Meyer Assessment (UL-FMA) [41], and National Institutes of Health Stroke Scale (NIHSS) were adopted to assess clinical functions.

### 2.7. Intervention

**Figure 2:**
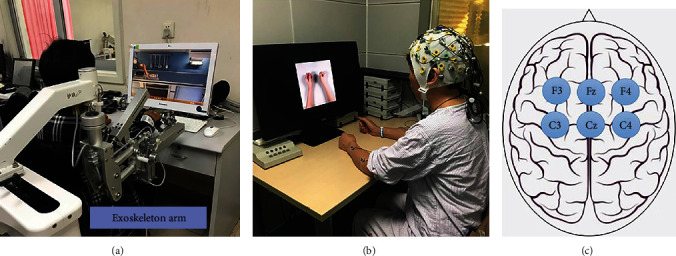
Diagrams of the experimental setup.

The nonimmersive VR intervention was delivered through an interactive training system (A2, YiKang Ltd., China) which comprised of a computer screen to display the virtual environment and a passive weight support exoskeleton arm. In a nonimmersive VR system, users interact with the virtual environment on a computer screen [42] with the exoskeleton arm as an interface. During VR intervention, participants were seated in front of a table facing the monitor, with the arms placed on the exoskeletal weight support.  Figure 2(a) illustrates the setting of the VR system. The exoskeleton arm support provided passive weight support at the elbow and wrist joints. A grip sensor was embedded in the handle bar to detect the application of grip force. The VR training tasks were provided to train the motion of reaching and reach to grasp. These tasks were as follows: (1) fried egg, (2) apple picking, and (3) archery. The tasks of fried egg and apple picking involved the users to first reach out to a virtual object, grasp the virtual object, and then transfer it to a designated area. Both of these tasks were designed for grasping and finger movement training. For the archery task, participants were asked to grip and maneuverer the virtual arrow to aim the target, followed by the release of the virtual arrow. This training item was designed for the training of pinching motion. The training for each task lasted for 15 minutes, giving a total intervention time of 45 minutes. Participants in the control group received conventional occupational therapy that consisted of task-orientated motor training, including grip strength, selective finger movement, and activities of daily living. Training frequency was matched to that in the VR group.

### 2.8. ERP Paradigm

The ERP experimental procedure and paradigms adopted in the present study were in accordance with a published study [31]. Participants were seated in front of a table in an electrically shielded laboratory with shoulders positioned between 0 and 10° flexion, elbows at 130° flexion, and wrists orientated in a neutral position. The motion of palm opening and closing of the hand occurred in the horizontal plane.  Figure 2(b) illustrates the starting position of the experiment. The procedure of the experiment was first explained to all participants verbally and was followed by 5 to 10 minutes of practice to familiarise with the protocol. Signal recording then began.

**Figure 3:**
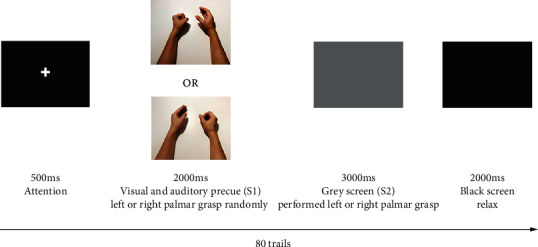
The ERP experiment paradigm.

Figure 2(c) illustrates the locations of the EEG electrodes that record the regions of interest during simultaneous recording of EEG and EMG. The ERP paradigm was as follows: a white fixation point “+” first appeared in the centre of the screen for 500 ms. Then, visual and auditory cues (S1) were given simultaneously for 2000 ms. A picture cueing grasp motion with either the left or right hand was displayed on the screen, accompanied by an auditory cue to indicate right or left side palmar grasp. During S1, the participants were required to judge the palmar grasp task. Then, a grey reaction window (S2) of 3000 ms appeared; the participants performed left or right palmar grasp and avoid making compensatory movements. Then, a dark screen of 2000 ms appeared; the participants restored their fingers and then entered the next trial. The experiment consisted of 40 trials for each hand, totalling 80 trials. The order of the trials was randomized.  Figure 3 illustrates the diagram of the ERP paradigm.

### 2.9. EEG

EEG activities were recorded by a 32-channel QuickAmp amplifier and Ag/AgCl scalp electrodes (Brain Products, Germany). The electrodes were positioned in accordance with the international 10-20 system. Electrodes were filled with conductive gel to maintain the impedance below 5 k*Ω* EEG. EMG signals were recorded in DC mode and sampled at 1000 Hz synchronized with event markers.

#### 2.9.1. Regions of Interest

Six electrodes of interest related to motor function were extracted for EEG analysis (left hemisphere: F3 and C3, right hemisphere: F4 and C4, and midline region: Fz and Cz). The F3, F4, and Fz lie over the premotor cortex, and the C3, C4, and Cz lie over the primary motor cortex. For the purpose of statistical comparison, the left and right side hemispheres were flipped right to left in the participant with a right hemispheric lesion so that the “left” hemisphere was always the lesioned hemisphere [22].

### 2.10. EMG

**Figure 4:**
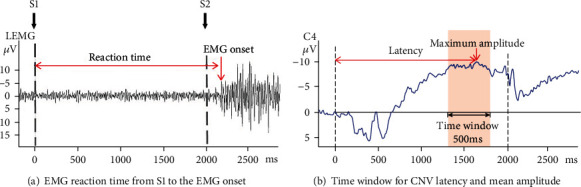
Timing window for signal processing data.

The EMG activities were simultaneously recorded with EEG. Two surface electrodes were placed along the extensor digitorum using 2 surface electrodes with a 2 cm interelectrode distance. The EMG reaction time was measured with the time from S1 to the EMG onset as shown in Figure 4(a).

### 2.11. Signal Processing

EEG signals were referenced to the bilateral mastoid. Eye movement artefacts were removed through the Ocular Correction Independent Component Analysis (ICA) as part of the standard operating procedure [14]. EEG and EMG signals were filtered using a 50 Hz notch filter and a bandpass filter from 0.1 to 30 Hz. In order to acquire stimulus-locked ERPs, the EEG and EMG signals were segmented into epochs of 500 ms pre- to 3000 ms post aligned to S1. The baseline was corrected according to the first 200 ms of the epochs, which was the 200 ms time window before the S1 onset.

The EMG reaction time was measured with the time from S1 to the EMG onset. As the maximum CNV amplitude was detected between 1300 ms and 1800 ms after S1, peak detection was used to detect the maximum CNV amplitude in this time window. The maximum amplitude must be higher than the amplitude of 2-3 points on the front and back sides [43]. The CNV latency was calculated as the time from S1 to the maximum amplitude. The mean CNV amplitude was calculated from 1300 to 1800 ms to acquire the average amplitude of the CNV potential (Figure 4(b)).

### 2.12. Statistical Analysis

Statistical analyses were performed using SPSS version 22 (IBM SPSS Statistics version 20, USA). The Wilcoxon signed-rank test was applied to verify the statistical significance of the changes in the ARAT, UL-FMA, and NIHSS within groups, and the Mann-Whitney *U* test was conducted to compare between the two groups. For EMG reaction time and CNV latency analyses, mixed model analysis of variance (ANOVA) was calculated, with TASK (paretic hand movement vs. nonparetic hand movement) and TIME (baseline vs. posttraining) as within-subject factors and GROUP (VR group vs. control group) as the between-subject factor. Mixed ANOVA with TASK (paretic hand movement vs. nonparetic hand movement), ELECTRODE (F3, C3, F4, C4, Fz, and Cz), and TIME (baseline vs. posttraining) as the within-subject factor and GROUP (VR group vs. control group) as the between-subject factor was performed to assess the training effects. The Greenhouse-Geisser adjustment was applied to adjust the degrees of freedom if the assumption of Mauchly's test of sphericity was not significant. Separate mixed model ANOVAs were tested for each level of electrodes. If there was a significant interaction in the within-subject factor and between-subject factor, then subsequent independent sample *t*-tests were performed to further investigate the differences between the two groups. The significance level for all statistical analyses was set at 0.05. Bonferroni-adjusted significance tests were performed to correct the *p* values of electrodes for multiple comparisons. Thus, the corrected significance level for the electrode was *α* = 0.05 ÷ 6 = 0.008 [44].

## 3. Results

### 3.1. Demographics

A total of 40 patients were screened, and 4 patients were excluded. The final sample population included 36 participants with upper limb hemiplegia resulting from the first occurrence of stroke. All enrolled participants completed the study, and the number of participants included in the analysis was as planned. Eighteen participants were allocated to the VR group, and 18 participants were allocated to the control group.  Table 1 gives a summary of the demographic data and clinical characteristics of the sample cohorts. The independent sample *t*-test showed no significant difference in the number of cases, sex, and age between the VR group and the control group (*p* > 0.05). The Mann-Whitney *U* tests showed no significant difference in the UL-FMA, ARAT, and NIHSS scores between the two groups before treatment (*p* > 0.05).

### 3.2. Behavioural Data

#### 3.2.1. EMG Reaction Time

The mixed model ANOVA indicated a significant main effect of TIME (*F*(1, 34) = 483.326, *p* ≤ 0.001) and a significant interaction between TIME and GROUP (*F*(1, 34) = 5.680, *p* = 0.023), as well as TIME and TASK (*F*(1, 34) = 14.943, *p* ≤ 0.001). Subsidiary analysis showed that the EMG onset time of the nonparetic hand task was significantly earlier than that of the paretic hand task in both groups before treatment (*F*(1, 34) = 21.099, *p* ≤ 0.001), whereas the two groups did not differ significantly of the nonparetic or the paretic hand task (*F*(1, 34) = 0.339, *p* = 0.564). After treatment, there was a significant main effect of TASK (*F*(1, 34) = 5.286, *p* = 0.028); the EMG onset time of the nonparetic and the paretic hand task in both groups was earlier than baseline (all *p* ≤ 0.001). In addition, the EMG onset time of the paretic hand in the VR group was earlier than that observed in the control group (*t* = 2.174, *p* = 0.04). There is no significant difference in the EMG onset time of the nonparetic hand between the two groups (*t* = 1.547, *p* = 0.13).  Table 2 presents the comparison of the EMG onset time before and after intervention for both groups.

#### 3.2.2. CNV Latency

The mixed model ANOVA conducted on the CNV latency revealed a significant main effect of TIME (*F*(1, 34) = 339.54, *p* < 0.001) and a significant interaction between TIME and GROUP (*F*(1, 34) = 4.855, *p* = 0.034). Subsidiary analysis showed that at baseline, the main effect of GROUP (*F*(1, 34) = 2.014, *p* = 0.165) and the interaction between GROUP and TASK (*F*(1, 34) = 0.013, *p* = 0.911) did not reach significance, which indicated that there was no significant intergroup difference in the CNV latency at baseline. After treatment, a significant main effect of TASK (*F*(1, 34) = 5.286, *p* = 0.028) was observed. Subsequent analysis revealed that the CNV latency of the paretic and nonparetic hand in both groups were earlier than baseline (VR group: paretic hand: *t* = 8.040, *p* ≤ 0.001, nonparetic hand: *t* = 11.775, *p* ≤ 0.001; control group: paretic hand: *t* = 11.886, *p* < 0.001, nonparetic hand: *t* = 11.106, *p* ≤ 0.001). In addition, the change in the CNV latency of the paretic hand after treatment was better in the VR group than in the control group (*t* = 2.411, *p* = 0.021), whereas the change of the nonparetic hand between the two groups did not differ (*t* = 1.151, *p* = 0.258).  Table 3 presents the comparison of CNV latency before and after intervention for both groups during nonparetic and paretic hand movements.

### 3.3. CNV Amplitude

**Figure 5:**
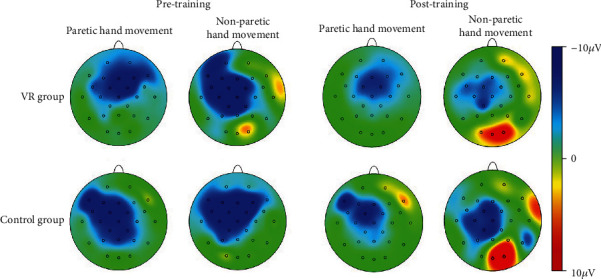
Topographic maps of participants in the two groups when executing paretic and nonparetic hand movement tasks.

**Figure 6:**
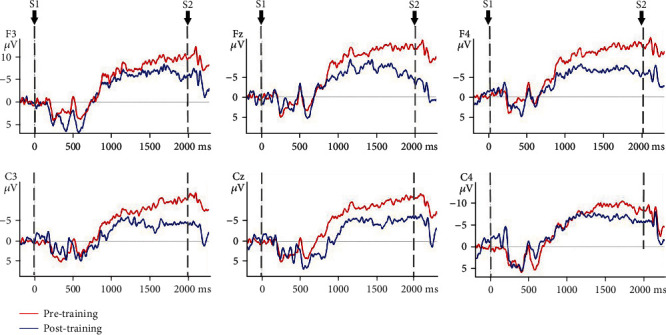
CNV amplitudes of the VR group during paretic hand movement. Negative is plotted upwards. Note: the 200 ms epoch prior to the S1 onset was the baseline CNV amplitude. The onset is at 0 ms point and is the S1 trigger time.

**Figure 7:**
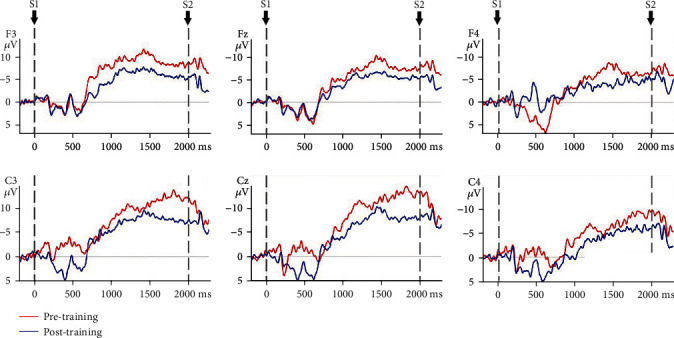
CNV amplitudes of the VR group during nonparetic hand movement. Note: negative is plotted upwards. The 200 ms epoch prior to the S1 onset was the baseline CNV amplitude. The onset is at 0 ms point and is the S1 trigger time.

**Figure 8:**
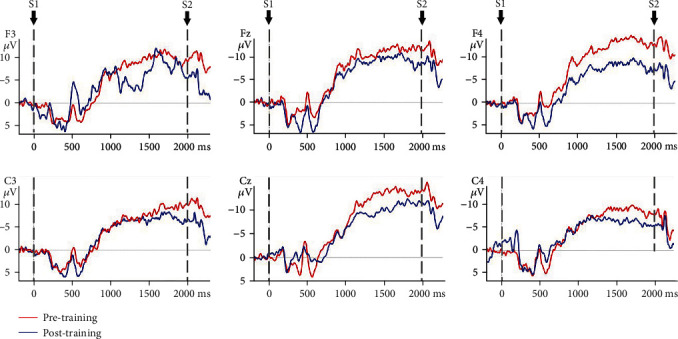
CNV amplitudes of the control group during paretic hand movement. Note: negative is plotted upwards. The 200 ms epoch prior to the S1 onset was the baseline CNV amplitude. The onset is at 0 ms point and is the S1 trigger time.

**Figure 9:**
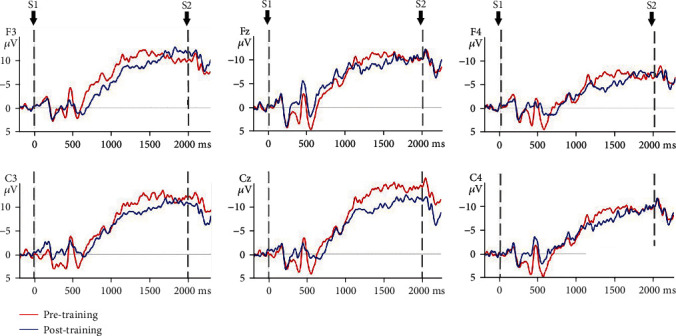
CNV amplitudes of the control group during nonparetic hand movement. Note: negative is plotted upwards. The 200 ms epoch prior to the S1 onset was the baseline CNV amplitude. The onset is at 0 ms point and is the S1 trigger time.

The mixed model ANOVA on the CNV amplitude between the VR group and control group of nonparetic and paretic hand tasks revealed a significant interaction between TASK, TIME, GROUP, and ELECTRODE (*F*(5, 170) = 6.915, *p* ≤ 0.001), significant interaction between GROUP and TIME (*F*(1, 34) = 156.167, *p* ≤ 0.001), and significant interaction between TIME and ELECTRODE (*F*(5, 170) = 4.383, *p* = 0.01). The CNV amplitude was modulated by TIME condition (*F*(1, 34) = 501.293, *p* ≤ 0.001) and ELECTRODE condition (*F*(5, 170) = 6.063, *p* ≤ 0.001). Therefore, TIME, GROUP, ELECTRODE, and TASK were tested for individual effects. At baseline, the interaction between TASK, GROUP, and ELECTRODE was not significant (*F*(1, 34) = 0.267, *p* = 0.609), which indicated that there was no significant difference in CNV amplitude of the nonparetic and paretic hand between the VR group and control group before treatment. After treatment, significant main effects of GROUP (*F*(1, 34) = 3.393, *p* = 0.02) on CNV amplitude were observed, which indicated significant improvement in CNV amplitude during nonparetic hand and paretic hand movement in both groups after treatment (*p* ≤ 0.001). Further investigation revealed that there was a significant main effect of TIME on the paretic (*F*(1, 34) = 2.744, *p* = 0.033) and nonparetic hand (*F*(1, 34) = 5.341, *p* ≤ 0.001) in the VR group and on the paretic (*F*(1, 34) = 7.803, *p* ≤ 0.001) and nonparetic hand (*F*(1, 34) = 7.944, *p* ≤ 0.001) in the control group. Then, the reduction in CNV amplitude between the VR group and control group after treatment was analysed. There was a significant interaction between TASK, GROUP, and ELECTRODE (*F*(5, 170) = 3.560, *p* = 0.03). The reduction in CNV amplitude in the VR group was better than that in the control group (*p* ≤ 0.001) when executing paretic hand movement, while the reduction in CNV amplitude was better in the VR group than in the control group (*p* ≤ 0.001) when executing nonparetic hand movement.  Table 4 presents the changes in CNV amplitude in the VR and control groups during nonparetic hand and paretic hand movements.  Figure 5 shows the topographic maps of participants in two groups when executing paretic and nonparetic hand movement tasks. Figures 6–9 give the graphical representations of CNV amplitudes for the VR and control groups during paretic and nonparetic hand movement.

### 3.4. Clinical Functions

The scores of UL-FMA, ARAT, and NIHSS improved in both groups after intervention. The improvements in ARAT and UL-FMA in the VR group were significantly higher than those in the control group (UL-FMA: *Z* = −2.338, *p* = 0.02; ARAT: *Z* = −2.088, *p* = 0.04). No significant difference in the reduction in NIHSS was observed between the VR and control groups (*Z* = −1.801, *p* = 0.07).  Table 5 presents a summary of the changes in NIHSS, UL-FMA, and ARAT in the VR group and control group.

## 4. Discussion

The present study investigated the impact of VR intervention on motor anticipation and upper limb function in patients with subacute stroke. One of the main findings was that EMG response time was significantly shorter in the VR group than in the control group during paretic hand movement postintervention. The reduction of CNV latency and peak amplitude in the VR group was significantly more than that in the control group. The improvements of upper limb motor function were significantly higher in the VR group than in the control group after treatment.

### 4.1. EMG Onset and CNV Latency

The reaction time of EMG is attributed to the effect of muscle onset, whereas CNV latency is related to the brain's computational demand and cognitive processing speed when executing a movement [45–47]. Impairments in motor anticipation among subacute stroke patients could be manifested as a delay in EMG reaction time and increased preparation time of cerebral hemispheres. This is supported by neural electrophysiological study which indicated a significant increase in cortical preparatory activation correlated with a behavioural enhancement [48]. In this study, the EMG response time and CNV latency decreased after intervention in both groups, which indicated that the EMG onset was earlier and the motor anticipation time of bilateral cerebral hemispheres was shortened in both groups. Previous studies reported that subacute stroke patients demonstrated a typical response-priming effect, with extended reaction time and CNV latency [22, 31]. The shorter reaction time might be related to the improvement in the neurocognitive process to plan an upcoming task. The larger reduction in reaction time in the VR group than that observed in the control group during paretic hand movement posttraining suggested that VR intervention may be more effective than conventional therapy training to improve the cognitive neural process. Task-oriented VR training requires the user to have an understanding of the upcoming task to be able to complete the task which in theory is able to improve cognitive function. The reduction in EMG reaction time and CNV latency tended to approach normal level postintervention, indicating an improvement in neural activation efficiency of motor anticipation and the improvement of motor dynamics of upper limb function [49]. Therefore, VR training may improve hemiplegic upper limb motor anticipation by promoting neuronal activation in stroke patients. This finding is consistent with a published study that investigated the cortical latency and central motor conduction time in stroke patients who underwent VR intervention [50] when assessed by transcranial magnetic stimulation. As with the finding of the present study, the improvement in cortical latency and central motor conduction time was significantly higher in the intervention group than in the control group. The potential mechanism underpins that the improvement in cognitive neural function is the reaction of brain neurotransmitters pathways, including cholinergic and dopaminergic pathways [51]. These provide further support that VR intervention may be superior to the control therapy in improving cognitive neural function.

### 4.2. CNV Amplitude

CNV amplitude is an index of anticipation and reflects the amount of cognitive neural resource [52] required to plan an upcoming task [29, 53]. Early literature reported cerebral activity of the contralesional hemisphere in the early stage of stroke during movement of the paretic side [54, 55]. A published study reported that subacute stroke patients recruit additional neural resources from the contralesional hemisphere during the motor planning phase of grasp motion [27, 31], as indicated by an increase in CNV amplitude in the lesional and contralesional hemispheres during paretic hand movement. Our previous EEG study indicated significant increase in cortical activity at contralesional C4 and ipsilesional C3 electrodes during nonparetic hand movement in patients with stroke [31]. The increase in bilateral cortical activity is related to the compensatory overactivation of the contralesional hemisphere and the lack of cross hemispheric inhibition from the lesional to nonlesional hemisphere. The present study observed significantly larger reduction in CNV amplitude in the midline and contralesional and ipsilateral area during nonparetic hand movement and paretic hand movement in the VR group than in control group. The reduction in CNV amplitude corresponds to the improvement in upper limb function which provides further support that VR intervention may be effective in reducing interhemispheric misbalance compared to conventional training. The reduction in CNV amplitude postintervention is also consistent with MRI studies suggesting that neuroplasticity recovery manifested as a decrease in sensorimotor cortex activation on the contralesional hemisphere [54], which can be interpreted as an increase in the efficiency of neural activation. Correlation between the recovery of motor function and relateralization of activation in the contralateral hemisphere was also reported [56]. Thus, the reorganization of motor control occurs after stroke and may involve the ipsilateral or contralateral cortex, depending on the location and size of the brain lesion and theoretically on the somatotopic organization of the residual pyramidal tracts [57]. The observed significantly larger reduction of CNV amplitude in the VR group during paretic hand movement provides further support that VR intervention may be effective in reducing hyperactivity of the contralesional hemisphere. The finding is given further support by another study that reported a reduction in the lateral index and the decrease in excessive dominance of the contralateral hemisphere post-VR intervention. Therefore, this study further supports that VR intervention may be more effective in reducing hemispheric lateralization compared to conventional occupational therapy [49].

### 4.3. Upper Limb Function

Both of the ARAT and UL-FMA demonstrated significant improvements in both VR and conventional therapy groups. The improvements in both ARAT and FMA-UA were significantly higher in the VR group than in the control group. These findings indicated that VR training may be superior to the conventional therapy in promoting motor function of the upper limb. The improvement in upper limb function induced by VR intervention is consistent with the results reported in a systematic review of a small to medium effect in favor of VR intervention when compared with conventional therapy [8]. Despite the statistical significant difference observed between the VR and conventional therapy groups, the actual between-group differences in UL-FMA and ARAT were small and less than the minimal clinically important difference [58, 59]. A Cochrane review stated that studies that reported superior outcome of VR tended to adopt VR as an augmentation to usual dosage of therapy where participants received more treatment time than the control group [33]. This study adopted matched intervention time in both groups which may be a potential reason for the observed small difference.

### 4.4. Limitations

The data in the present study should be interpreted with caution due to its limitations. The study included participants at the subacute stage of stroke which limits the external validity of the finding. Future research should include participants at the acute and chronic stage of stroke to enhance the external validity of the findings. This study recruited participants who were between 40 and 80 years old. The wide age range limits the generalizability of the study findings. Further study that includes smaller age range of the sample population is recommended to substantiate the findings of the present study. The right to left flipping of the scalp site in participants with the right hemisphere lesion site may be considered a limitation. However, it is not uncommon in published literature that involves either EEG or fMRI study to perform the flipping as a means to increase statistical power [60]. The present study did not involve a follow-up period, and it remains unclear if the observed benefit may carry on in the longer term.

## 5. Conclusions

VR intervention is superior to conventional therapy in improving the cognitive neural process of motor anticipation and reducing the excessive compensatory activation of the contralesional hemisphere. The improvements observed in the cognitive neural process corroborated with the improvements in hand function. No firm conclusion could be drawn if VR intervention is superior to conventional therapy in promoting hand function recovery due to the nonclinical significant difference observed between the two groups.

## Figures and Tables

**Table 1:** Demographics and clinical characteristics of the sample cohorts. UL-FMA: Upper Limb Fugl-Meyer Assessment; ARAT: Action Research Arm Test; NIHSS: National Institutes of Health Stroke Scale; MMSE: Mini-Mental State Examination.

	VR group (*n* = 18)	Control group (*n* = 18)
Age (SD), y	57.8 (8.4)	58.4 (9.3)
Sex		
Male	10	10
Female	8	8
UL-FMA	26.33 (6.04)	26.00 (7.09)
ARAT	18.55 (5.97)	18.33 (5.17)
NIHSS	5.44 (2.91)	5.39 (2.85)
MMSE	26.05 (2.64)	25.61 (2.87)

**Table 2:** Comparison of EMG onset time before and after intervention during nonparetic and paretic hand movements.

Group	Pre (ms)	Post (ms)	*t*	*p*
Nonparetic hand				
VR group	2419.71 ± 95.14	2378.93 ± 98.73	10.27	≤0.001
Control group	2449.56 ± 122.27	2418.75 ± 114.92	6.08	≤0.001
Paretic hand				
VR group	2526.3 ± 121.78	2472.06 ± 117.28	15.77	≤0.001
Control group	2532.19 ± 122.40	2486.56 ± 121.75	23.05	≤0.001

**Table 3:** Comparison of CNV latency before and after intervention during nonparetic and paretic hand movements.

Group	Pre (ms)	Post (ms)	In change (ms)	*t*	*p*
Nonparetic hand					
VR group	1661.33 ± 134.48	1553.00 ± 117.88	106.06 ± 54.39	11.78	≤0.001
Control group	1654.56 ± 197.50	1565.94 ± 197.77	88.61 ± 30.74	11.11	≤0.001
*p*			0.26	1.15	
Paretic hand					
VR group	1712.33 ± 143.99	1593.39 ± 134.39	111.89 ± 39.18	8.04	≤0.001
Control group	1714.33 ± 191.95	1631.56 ± 193.41	82.78 ± 30.73	11.89	≤0.001
*p*			0.02	2.41	

**Table 4:** Changes in CNV amplitude in the VR and control groups during nonparetic hand and paretic hand movements.

Nonparetic hand movement (mV)				Paretic hand movement (mV)		
Electrode	VR group	Control group	*p*	VR group	Control group	*p*
C3	2.06 ± 0.49	1.16 ± 0.26	≤0.001	1.84 ± 0.52	1.21 ± 0.37	≤0.001
Cz	2.11 ± 0.40	1.35 ± 0.27	≤0.001	2.18 ± 1.18	1.29 ± 0.24	0.01
C4	1.96 ± 0.34	1.31 ± 0.21	≤0.001	1.91 ± 0.58	1.08 ± 0.24	≤0.001
F3	1.37 ± 0.62	1.19 ± 0.19	0.26	1.92 ± 0.52	1.24 ± 0.19	≤0.001
Fz	2.27 ± 0.87	1.23 ± 0.31	≤0.001	2.09 ± 0.50	1.37 ± 0.30	≤0.001
F4	2.06 ± 0.94	1.20 ± 0.24	≤0.001	2.04 ± 0.71	1.05 ± 0.30	≤0.001

**Table 5:** A summary of the changes in NIHSS, UL-FMA, and ARAT in the VR group and control group.

Group	UL-FMA	ARAT	NIHSS
VR group	4.72 ± 0.87	5.06 ± 0.91	2.17 ± 0.83
Control group	3.89 ± 1.15	4.11 ± 1.20	1.83 ± 0.50
*Z*	-2.338	-2.09	-1.80
*p*	0.02	0.04	0.07

## Data Availability

The data that support the findings of this study are available from the First Affiliated Hospital, Sun Yat-sen University, but restrictions apply to the availability of these data, which were used under license for the current study and are not publicly available. Data are however available from the authors upon reasonable request and with permission of the First Affiliated Hospital, Sun Yat-sen University.
